# Radar-Based Detection of Respiration Rate with Adaptive Harmonic Quefrency Selection

**DOI:** 10.3390/s20061607

**Published:** 2020-03-13

**Authors:** JeeEun Lee, Sun K. Yoo

**Affiliations:** 1 Graduate Program of Biomedical Engineering, Yonsei University, Seoul 03722, Korea; jeunlee@yuhs.ac; 2 Department of Medical Engineering, Yonsei University College of Medicine, Seoul 03722, Korea

**Keywords:** respiration rate, radar, quefrency

## Abstract

Continuous respiration monitoring is important for predicting a potential disease. Due to respiration measurements using contact sensors, it is difficult to achieve continuous measurement because the sensors are inconvenient to attach. In this study, a radar sensor was used for non-contact respiration measurements. The radar sensor had a high precision and could even be used in the dark. It could also be used continuously regardless of time and place. The radar sensor relied on the periodicity of respiration to detect the respiration rate. A respiration adaptive interval was set and the respiration rate was detected through harmonic quefrency selection. As a result, it was confirmed that there was no difference between the respiratory rate measured using a respiration belt and the respiratory rate detected using a radar sensor. Furthermore, case studies on changes in the radar position and about measurement for long periods confirmed that the radar sensor could detect respiration rate continuously regardless of the position and measurement duration.

## 1. Introduction

Respiration is a bio-signal that is useful for monitoring patients and detecting a potential disease. Therefore, it is essential to be able to track continuously and measure the respiration rate. The continuous monitoring of respiration could serve as an early warning in high-risk situations. Respiration can be measured using two types of methods: contact methods, in which there is an instrument attached directly to a subject’s body, and non-contact methods, in which the instrument is not attached. The contact methods include the use of a belt on the chest or abdomen to detect body movement and the use of a nasal pressure transducer sensor to measure airflow [1,2]. Although the contact method has the advantage of greater accuracy, it is difficult to use continuously due to its inconvenience. Therefore, the development of various sensor technologies has occurred in many studies on non-contact methods.

Radar, optical, and thermal sensors are the most commonly used kinds of sensors for non-contact measurement [3]. In particular, with the recent development of the mobile environment, non-contact sensors are capable of monitoring respiration in a variety of situations. In other words, it is now possible to use non-contact sensors to detect and manage a variety of medical situations that could occur in daily life, such as sleep apnea and respiratory failure [4]. Radar sensors use a technique by which a distance is mapped using the time taken for a signal to reach an object, be reflected, and return to the sensor. Moreover, because such sensors can obtain signals at low cost, using low power, and with high precision, they could be used as substitutes for other sensors, such as cameras. Radar sensors, in particular, have the major advantage that they can be used in dark places, such that they can be used continuously, regardless of time and place [5].

A radar sensor has a high degree of precision but is highly sensitive to the external environment. Noise from the external environment can change the radar sensor output. Therefore, in previous studies on radar sensors, multiple radars were used to correct the phase [6]. However, when using multiple radars, it is necessary to detect their positions, which requires additional space and cost, and complicates the computation process. In addition, to reduce the changes in phase, the signal is detected using a spectrum analysis. However, even with this, it is still difficult to measure respiration precisely due to phase noises and harmonics [7]. In particular, when respiration is measured in the mobile environment, there can be a variety of noises caused by several environmental variables. To reduce such noise, it is critical to calculate respiration using its unique and inherent characteristics. 

The purpose of this study was to confirm the feasibility of measuring respiration rates using a radar sensor. Normal breathing has a periodic pattern, and the respiration rate falls within a certain range. Therefore, in this study, a respiration adaptive interval, showing unique characteristics of respiration, was set to detect the respiration rate. Moreover, harmonic frequency selection that reflects information about the respiratory cycle was done to detect the respiration rate. The respiration rate was calculated using the proposed method was compared with the respiration rate measured from a respiration belt used as the ground truth.

## 2. Methods

### 2.1. Experiment

#### 2.1.1. Experiment Overview

Sixteen adult males without any physical or mental conditions were chosen to be monitored for this study (sex: male, age: 25.1 ± 1.3, body mass index (BMI): 22.54 ± 2.02, chest circumference 90.4 ± 4.9). The room dimension was approximately 10 m^2^. The distance between the radar sensor and the subject was approximately 1 m. The radar signal was acquired using a radar module (LG Innoteks, Seoul, Republic of Korea). The subjects, each of which had filled out a written consent form prior to this study, had their respiration measured for 20 min. For the measurement, each subject was requested to lie on a bed, and the respiration data were acquired from a radar at a sampling rate of 500 Hz. There were no additional people in the room during data acquisition. For comparison with the respiration signals from the radar, each subject wore a respiration belt at chest level to provide a measure of respiration that served as a ground truth signal. The belt used was a MP 150TM (BIOPAC Systems, Inc., Goleta, CA, USA), which sampled at the rate of 500 Hz.

#### 2.1.2. Case Studies

The radar sensor used for these non-contact measurements could also be applied as part of a mobile system. Therefore, two case studies were performed to confirm the feasibility of using a radar sensor.

In the first case study, the purpose was to analyze the effectiveness of a radar sensor in relation to its location. Using a radar sensor in a non-medical environment (such as a home) changes the placement of the radar, depending on the person taking measurements and the space. The experimental conditions were the same as in the previous experiments except one more radar was added. The new radar was located to one side of the subject (within 1 m) and worked simultaneously with the default radar to acquire data from 10 people.

**Figure 1:**
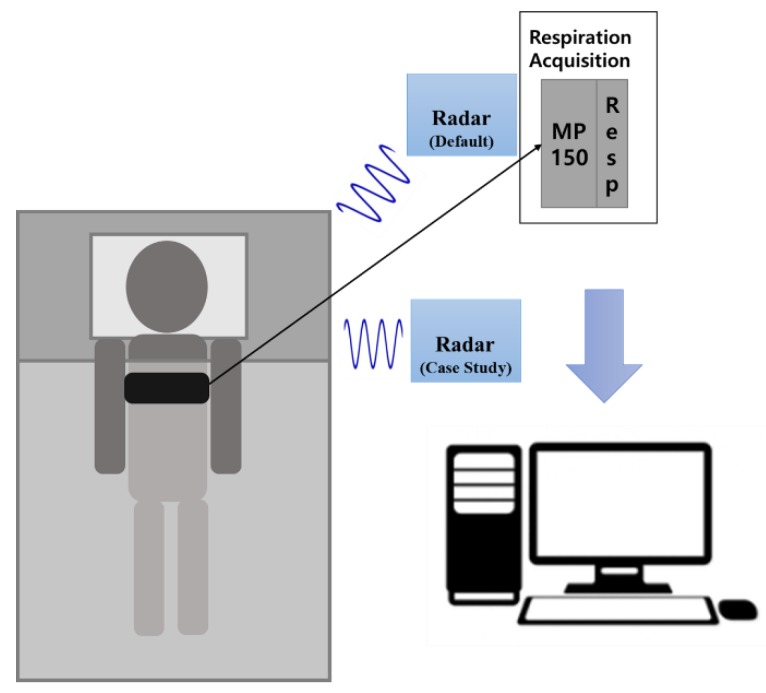
Experimental setup.

The second case study was conducted to evaluate the respiration rate detection performance over a long period. The radar sensor could be used for measurement over long intervals without any inconvenience because this was a non-contact measurement. The experimental conditions were the same as in the existing experiments. The data were acquired while a subject was sleeping to minimize non-respiratory movement for a long time. Figure 1 shows the experimental setup. The default radar was located above the subject and the second radar added for the case study was located beside the subject. The respiration belt was attached at chest level.

### 2.2. Adaptive Harmonic Quefrency Selection

To remove the noise and instability of the radar sensor and extract only respiration signals, a 0.4-Hz low-pass filter (LPF) was designed to limit the signal bandwidth. 

Respiration can be expressed as a quasi-periodic signal, as given in Equation (1). The term Resp(t) is the respiration signal at time t, and T is the cycle of a single breath. The period of each single breath is an unpredictable factor, therefore it is expressed as λT.

(1) Resp(t)=Resp(t+λT)

In this study, the acquired signal was processed into a 1-min epoch, as shown in Equations (2) and (3). Here, R(n) is the number of radar-sensor signals measured during the 1-min epoch; r is each sample obtained from the radar sensor; α(n) is the total sample before n minutes of processing, where n is in units of minutes; and SR is the sampling rate in samples per second (500 Hz in this study).

(2) α(n)=60×SR×(n−1)

(3) R(n)=[α(n)+r(1)α(n)+r(2)⋯α(n)+r(60×SR)]

Respiration is not perfectly periodic; rather it is quasi-periodic. Therefore, it has multiple harmonics for a respiration cycle T, and the cycle of a single breath changes continuously. In addition, when using the radar sensor, there are multiple frequency components due to environmental noise. To decompose the respiration signals intermingled with data from various noise sources, the radar sensor signals were converted into a frequency domain. After placing a hamming window on R(n), a fast Fourier transform (FFT) was used to convert them to the frequency domain. From Equation (4), the respiration cycle was detected by extracting the highest cepstrum component in the respiration-related frequency range. The term ℱ stands for the FFT and $C_{R}$ is the value of the inverse FFT of the radar sensor. This includes the cepstrum information [8,9].

(4) $$C_{R}={\left|{ℱ}^{-1}\left\{log\left({\left|ℱ\left\{R\left(n\right)\right\}\right|}^{2}\right)\right\}\right|}^{2}$$

**Figure 2:**
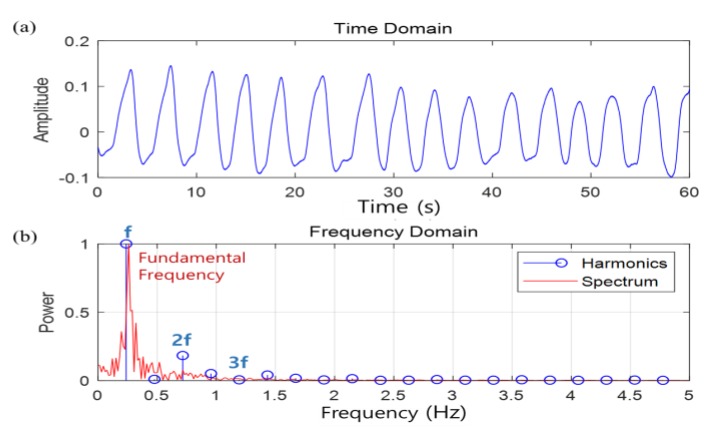
Harmonic component of the respiration signal: (**a**) time domain of respiration and (**b**) frequency domain of respiration.

Figure 2 shows the harmonic components of the respiration signal. Figure 2a shows quasi-periodicity as the respiration signal in the time domain, whereas Figure 2b shows the harmonic components in the frequency domain of respiration.

The peak extracted from $C_{R}$ shows that the components of the harmonics were periodic. The fundamental frequency is related to pitch and the frequency pitch can be estimated by using the quefrency at the point where the peak appears [10,11]. This can be calculated using Equation (5), where the term $QF_{\max\_peak}$ represents the quefrency of the point at which the maximum peak appears. In this study, to select the harmonic quefrency, the quefrency domain for finding the maximum peak was limited to the interval including the start point (S.P) and end point (E.P). If it is assumed that respiration usually occurs within the range 5–24 times in one minute [12], S.P and E.P can be calculated using Equations (6) and (7), respectively. Thus, the range that is adaptive to respiration is 1251–6000.

(5) $$Pitch=SR/\left(QF_{\max\_peak}\left[S.P–E.P\right]\right)$$

(6) S.P=SR/(24/60)+1

(7) E.P=SR/(5/60)

Finally, the respiratory rate, R.R (min^−1^), was calculated using Equation (8) by including the pitch extracted from the respiration domain.

(8) $$R.R\left(\min^{-1}\right)=Pitch\times 60$$

**Figure 3:**
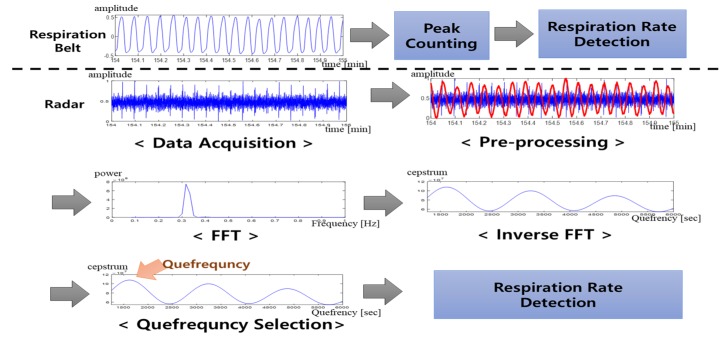
Overview of the respiration rate detection. FFT: Fast Fourier transform.

Figure 3 is an overview of the respiration rate detection method proposed in this study. The signal data were obtained from the subject using both the respiration belt and the radar sensor. A window without overlap was applied to the measured signal to calculate the respiration rate. The signals from the respiration belt were used to evaluate both the algorithm and the radar sensor. The signals from the radar sensor were processed using the 0.4-Hz LPF and then converted to the quefrency domain using inverse FFT. The respiration rate was detected in the respiration adaptive domain using the harmonic quefrency selection.

### 2.3. Evaluation

#### 2.3.1. Ground Truth

The respiration rate calculated from the respiration belt was used as ground truth for the evaluation of the performance of the radar sensor (see Section 3.2, Section 3.3 and Section 3.5). The respiration belt detects the respiration rate through contact measurement of the subject. Therefore, using the respiration belt, a respiratory waveform can be visually detected in the data without any pre-processing. The respiration rate was calculated by counting the peaks from the respiration belt, which were used as the ground truth. The threshold for detecting peaks in the respiration belt data was calculated using Equation (9). The term $R_{Gnd}\left(n\right)$ is the signal measured from the respiration belt. If the peak was larger than the threshold, it was detected, and the peak was counted only when the interval between the peaks was 2.5 s or more.

(9) $$\mathrm{Threshold}=0.8\times \left(1/\left(\max\left(R_{Gnd}\left(n\right)\right)-\min(R_{Gnd}\left(n\right)\right)\right))$$

#### 2.3.2. Evaluation of the Respiration Rate Comparison

One purpose of this study was to detect the respiration rate while achieving similar performance between the ground truth and radar sensor with adaptive harmonic quefrency selection (see Table 1, Table 2 and Table 3). Therefore, comparison of the detected respiration rates was used as the evaluation parameter. The peaks counted in the data coming from the respiration belt were used as the ground truth. When the difference between the ground truth and suggested method was small, the accuracy in the detection of the respiration rate was high.

#### 2.3.3. Statistical Evaluation

Statistical evaluation was conducted to confirm the reliability of the result. A Bland–Altman plot was used to evaluate the difference in the detected respiration rates of the ground truth and the proposed method (see Figures 5, 6 and 9). A Bland–Altman plot is a single regression analysis that uses the means of the estimated respiration rate and ground truth as independent variables, and the difference between the estimated respiration rate and ground truth as a dependent variable. This shows the bias of the measurement result based on the 95% confidence interval [13,14].

## 3. Results

### 3.1. Visual Inspection

**Figure 4:**
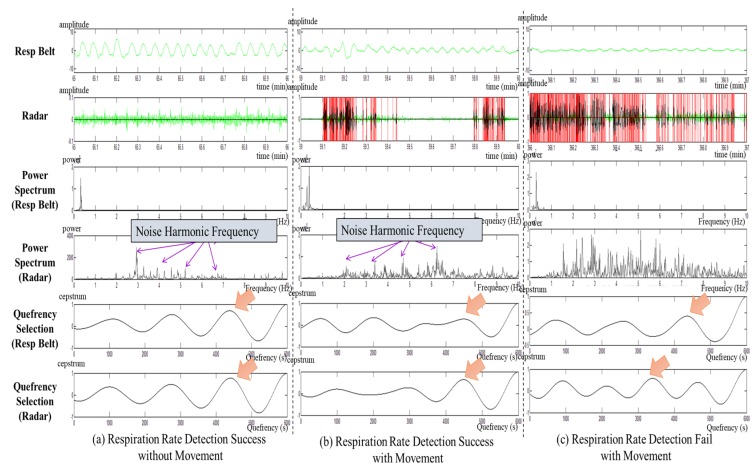
Visual inspection results with movement noise: (**a**) respiration rate detection success without movement, (**b**) respiration rate detection success with movement, and (**c**) respiration rate detection failure with movement.

Figure 4 shows the process of 1-min respiration rate detection using movement noises. The subjects made intentional movements to allow for comparison of the difference in respiration rate according to such movements. In Figure 4, (a) is a case of respiration rate detection when there was no non-respiratory movement; (b) is a case in which there was movement caused by occasional shaking of the body when detection of the respiration rate was successful; and (c) is a case in which detection of the respiration rate failed due to excessive noise, along with continuous body shaking. In Figure 4, the red line of the radar shows a domain with high energy due to a great deal of movement. As the movement noise increased in the power spectrum of the radar, the noise components increased such that the harmonic frequency also affected the respiration adaptive domain. In addition, if the quefrency selection points of the respiration belt and the radar were identified, they match when there was no or only a little movement. Moreover, when there were many movements, the quefrency selection point changed. If the noise signals were too strong, they also affected the respiration adaptive domain, and consequently, there was a gain in harmonic components. This made it difficult to detect the respiration rate.

### 3.2. Radar-Based Respiration Rate Detection

In this study, the respiration rate measured using peak counting in the respiration belt data was considered the ground truth. Table 1 shows the average respiration rate for each subject as measured using the respiration belt and using the radar. When the respiration belt was considered the ground truth, the difference in respiration rate was between 0 and 2 breaths per minute. There were some errors due to the sensitivity of the sensor. The radar data shows no significant difference in respiration rate detection performance compared to the data from the respiration belt, where the former was used with adaptive harmonic quefrency selection. Bland–Altman analysis showed that the mean difference ranged from 0.1 to 2.1 with an average bias of 0.6. The closer the mean difference was to 0, the smaller the difference between the two sensors. The result shows a difference of less than one breath per minute. Moreover, when the smallest confidence interval for the respiration rate was 1.3, the mean difference was also the smallest (−0.1 for subjects 1 and 2). When the widest confidence interval for the respiration rate was 4.70, the mean difference was −1.1, which was approximately one breath per minute (subject 13).

**Figure 5:**
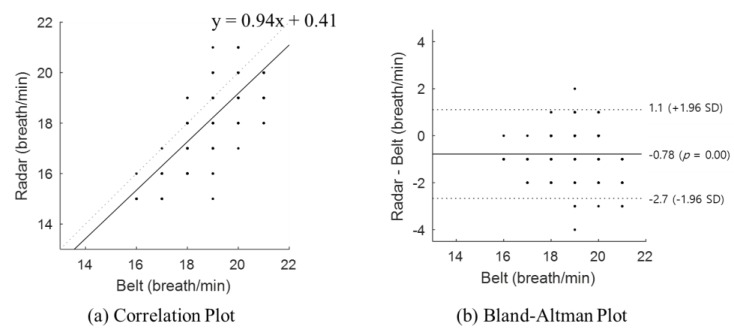
Statistical evaluation of the radar sensor according to all subjects: (**a**) correlation plot and (**b**) Bland–Altman plot.

Figure 5 shows the statistical results of the detected respiration rate from all subjects, where (a) shows the correlation plot and (b) shows the Bland–Altman plot. The two sensors used for the respiration rate detection showed a linear correlation. The Spearman rank-order correlation coefficient was 0.77 (*p* < 0.001) and it shows a positive correlation between the ground truth and radar sensor results. Moreover, the Bland–Altman plot indicates that the mean difference was 0.78, less than one breath per minute, and that the respiration rate was mostly at the 95% limits of agreement. The radar-sensor-estimated respiration rate could produce results ranging from a 1.1 breath/min overestimation to 2.7 breath/min underestimation.

### 3.3. Comparison of Respiration Rates

The respiration rate was detected by applying other methods to the radar signal acquired during the experiment. The other methods were peak counting and FFT-based spectrum peak extraction. Peak counting is a method of counting the peaks of a pre-processed radar signal. For peak detection, a threshold is calculated based on Equation (9). The FFT-based spectrum peak process transforms the radar signal to the frequency domain to extract a spectrum peak. The maximum peak that occurs in the frequency domain determines the respiration rate, assuming that the maximum peak of the frequency is related to respiration.

The respiration rate estimated using two different approaches (peak counting and FFT) were respectively compared with the ground truth (see Table 1, Belt). Table 2 gives the respiration rates using different approaches, the difference from the ground truth (Diff), and the Bland–Altman analysis results for each compared method. In the table, M.D. means mean difference, and 2SD indicates the 95% limit of agreement. Using peak counting, the difference in the respiration rate per minute could be as low as 0 and as high as 6. In addition, in the case of FFT-based spectrum peaks, the difference in respiration rate per minute was at least 0 and at most 5. Both methods showed low performance compared with adaptive harmonic quefrency selection. The Bland–Altman analysis showed that the mean differences were 1.7 and −1.5 for peak counting and FFT, respectively. When adaptive harmonic quefrency selection was used, the mean difference was close to 0. However, the other methods were > 1. Moreover, their 95% limits of agreement were wider than for the newly proposed method. This means that the respiration rates detected using the previous methods were not reliable. Therefore, the results show that adaptive harmonic quefrency selection had the best performance compared with those used in previous studies (see Table 1, Radar).

**Figure 6:**
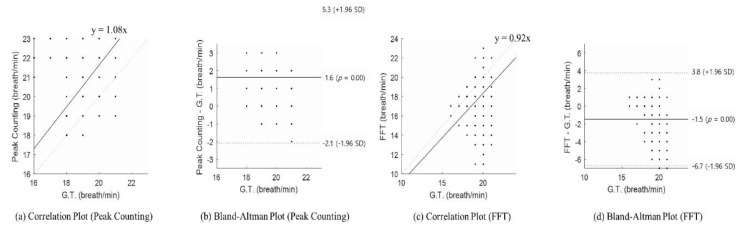
Statistical evaluation to compare other methods: (**a**) correlation plot of peak counting, (**b**) Bland–Altman plot of peak counting, (**c**) correlation plot of the FFT, and (**d**) Bland–Altman plot of the FFT. G.T.: Ground Truth.

Figure 6 shows the statistical results of the detected respiration rate with other methods (peak counting and FFT). Here, (a) is the correlation plot for peak counting, (b) is the Bland–Altman plot for peak counting, (c) is the correlation plot for FFT, and (d) is the Bland–Altman plot for the FFT results. For peak counting, the Spearman rank-order correlation coefficient was 0.21 (*p* < 0.001), which means a weak correlation. For FFT, it was not statistically significant (*p* > 0.05). As shown in the Bland–Altman plot, the bias of those methods was higher than that of the proposed method (average of peak counting: 1.6; average of FFT: −1.5; average of the proposed method: 0.29). The confidence interval of those methods also had a wider range than the proposed method did. The peak-counting-estimated respiration rate could produce results ranging from a 5.3 breath/min overestimation to a 2.1 breath/min underestimation. The FFT-estimated respiration rate could produce results ranging from a 3.8 breath/min overestimation to a 6.7 breath/min underestimation.

### 3.4. Radar Comparison

Table 3 is the result of the first case study. Radar 1 was the default radar and the radar 2 was the new radar (see Section 2.1.2). The ground truth (G.T.) was also measured using a respiration belt with peak counting. The respiration rate was detected using each radar with adaptive harmonic quefrency selection and they were respectively compared with the ground truth. Table 3 shows the average respiration rate of each subject measured using both radars and the ground truth. The difference in the respiration rate was 0–1 breaths per minute for each radar. It revealed that each radar sensor had low sensitivity to position when adaptive harmonic quefrency selection was used. The Bland–Altman analysis showed that the measurements were reliable through the mean difference and 95% limits of agreement. For radar 1, the mean difference ranged from 0 to 0.5 breath/min with an average of −0.1 breath/min as a bias. For radar 2, the mean difference ranged from 0.1 to 0.8 breath/min with an average of −0.2 breath/min as a bias. The result from each radar shows a difference of less than one breath per minute. The smallest confidence intervals for the respiration rate were 0.96 (subject 6) and 1.30 (subject 9) for radars 1 and 2, respectively. The widest confidence intervals for the respiration rate were 2.40 (subject 2) and 3.38 (subject 10) for radars 1 and 2, respectively.

**Figure 7:**
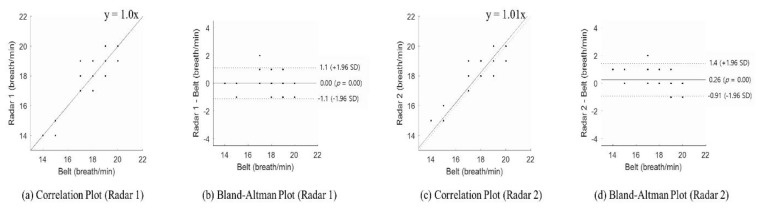
Statistical evaluation of both radar sensors: (**a**) correlation plot for radar 1, (**b**) Bland–Altman plot for radar 1, (**c**) correlation plot for radar 2, and (**d**) Bland–Altman plot for radar 2.

Figure 7 shows the statistical results of the detected respiration rate from the two radar sensors. In Figure 7, (a) and (c) show the correlation plot of each radar sensor, and (b) and (d) show a Bland–Altman plot of each radar sensor. The Spearman rank-order correlation coefficient of each radar sensor was 0.82 (*p* < 0.001) and 0.80 (*p* < 0.001) respectively. It shows that the ground truth and each radar sensor produced positive correlations. Therefore, the respiration rate rarely changed due to the radar positioning. The Bland–Altman plot indicated that the mean differences were 0 and 0.26, respectively, less than one breath per minute. Furthermore, the respiration rate was mostly at the 95% limits of agreement. The radar-1-estimated respiration rate could produce results ranging from a 1.1 breath/min overestimation to a 1.7 breath/min underestimation and the radar-2-estimated respiration rate could produce results ranging from a 1.4 breath/min overestimation to a 0.9 breath/min underestimation.

### 3.5. Respiration Rate Detection Over Long Periods

**Figure 8:**
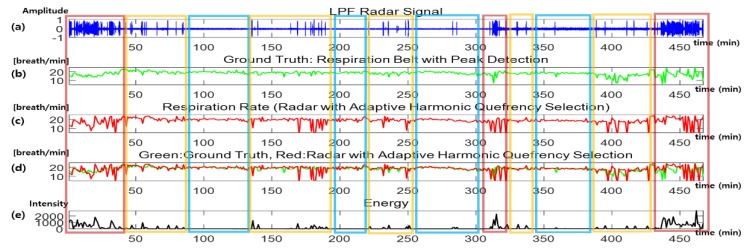
Respiration rate detection over long periods: (**a**) radar signal, (**b**) respiration rate from the respiration belt (ground truth), (**c**) respiration rate from the radar (proposed method), (**d**) comparison of the respiration rates from the respiration belt and radar signals, and (**e**) movement energy. LPF: Low-Pass Filter.

Figure 8 shows the sleep data for about 8 h where the x-axis shows the duration (min). In Figure 8, (a) shows the filtered data of the radar sensor, (b) and (c) show the respiration rates detected using each different sensor, (d) shows a comparison of the respiration rates, and (e) shows the energy extracted from the radar sensor. The energy extracted from the radar sensor was calculated using Equation (10) where N is the number of samples:

(10) $$\mathrm{Energy}=\frac{\sum_{1}^{N}{\left|R\left(n\right)\right|}^{2}}{N}.$$

The red box (first column on the left side) contains severe noise due to continuous body shaking; as a result, the respiration rate detection failed. The yellow boxes show results when the body only occasionally moved. In the blue boxes, there was no non-respiratory movement and the respiration rates were more accurate than in the other sections.

The larger the body movements, the higher their energy; as such, it was possible to estimate the movement state using an energy graph. From the data obtained at the beginning and 30 min from the end, the subject showed more movement at those times. Moreover, because of the body movements during sleep, the energy data in such sections were higher than in other sections. Therefore, from Figure 8, it can be seen that the respiration rate detected using the respiration belt was similar to that of the rates detected using radar, except in the high-energy sections. This means that it was possible to detect a respiration rate, even over a long period, using the data acquired with a radar sensor (nearly equivalent to the respiration belt results), if adaptive harmonic quefrency selection was applied.

**Figure 9:**
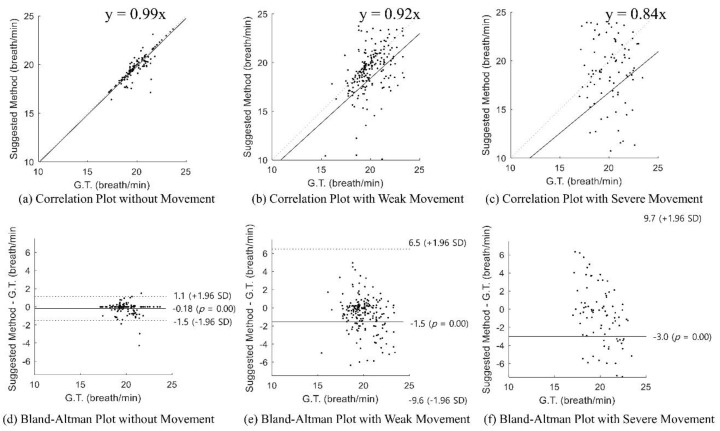
Statistical evaluation of the respiration rate detection over long periods: (**a**) correlation plot without movement, (**b**) correlation plot with weak movement, (**c**) correlation plot with severe movement, (**d**) Bland–Altman plot without movement, (**e**) Bland–Altman plot with weak movement, and (**f**) Bland–Altman plot with severe movement.

Figure 9 shows the statistical result of the detected respiration rate over long periods with respect to different levels of movement: (a)–(c) show the correlation plots and (d)–(f) show the Bland–Altman plots. Without movement, the Spearman rank-order correlation coefficient was 0.87 (*p* < 0.001) and it showed a strongly positive correlation between the ground truth and the radar sensor result (Figure 9a). Also, the Bland–Altman plot indicated that the mean difference was −0.18 and that the respiration rate was mostly at the 95% limits of agreement. The radar-sensor-estimated respiration rate could produce results from a 1.1 breath/min overestimation to a 1.5 breath/min underestimation (Figure 9d). With weak movement, the Spearman rank-order correlation coefficient was 0.43 (*p* < 0.05) and it showed a weak correlation between the ground truth and the radar sensor result (Figure 9b). Furthermore, as shown in the Bland–Altman plot, the mean difference was −1.5 but the 95% limit of agreement was wide due to movement error (Figure 9e). With severe movement, the results were not statistically significant (*p* > 0.05). As shown in the Bland–Altman plot, the mean difference was −3.0 and the 95% limit of agreement was the widest (Figure 9f). As shown in Figure 9, when the movement occurred, the detected respiration rate was not accurate.

## 4. Discussion

In this study, radar sensors were used to measure respiration as a non-contact method. In addition, adaptive harmonic quefrency selection was proposed to improve the results for of the detection of respiration rates using signals acquired from radar sensors. 

Respiration is a bio-signal that is directly related to an individual’s life. Clinicians predict clinical events by observing changes in the respiration of a patient and use it to identify degrees of risk. In particular, dyspnea is a common symptom in newborns and patients. Overall, 85% of dyspnea cases occur during asthma, pneumonia, myocardial ischemia, and lung disease [15]. In addition, the measurement of respiration is important because respiratory distress is induced by more than 30 diseases in a variety of organs, including the heart [16,17]. Clinicians use a patient monitor or a sensor to monitor a patient’s respiration [1]. However, it is not possible to use typical contact respiration monitoring methods to monitor patient respiration for follow-up once a patient has been discharged. Moreover, contact respiration monitoring methods have other issues: it is difficult to place the sensors on the body and to use them continuously [2]. Therefore, given these limitations, it would be important to develop non-contact sensors that able to cope with the limitations of contact respiration monitoring methods. 

A variety of sensors, such as radar, optical, and thermal sensors, are used to measure respiration without direct contact [3,4]. Respiration is estimated based on movement (expansion and contraction) of the lungs, which causes related movements of the exterior of the body. Radar sensors process reflected signals rapidly enough to detect such motions. Because radar is very accurate for the detection of object motion, it is outstanding at detecting respiration signals. Radar sensors have a high precision but are also sensitive to noise. Therefore, depending on the method used to process the acquired signals, the performance of the respiration rate detection varies. In particular, radar sensors respond to movement via a change in the signal phase. To resolve this issue, several studies have been done on the modeling of reflective signals from multiple radars and on spectral analysis [18]. In this study, the respiration rate was estimated using the adaptive quefrency domain. The harmonic quefrency selection of the fundamental frequency could be used to inversely estimate the respiration rates. 

The respiration of each subject was monitored for 20 min to monitor continuous respiration, in which the subject might have natural movements or a change in respiration rate. The acquired data were verified by comparing the respiration rate obtained through the use of peak counting (with adaptive harmonic quefrency selection) with the respiration rate obtained from the respiration belt as the baseline. Moreover, the respiration rate obtained using the respiration belt was compared with that of a radar sensor to evaluate the radar sensor’s respiration rate detection performance. As a result, it was possible to confirm that respiration rate detection can be done in a stable manner by applying adaptive harmonic quefrency selection to the signals obtained from the radar sensor. The non-contact method of respiration monitoring will soon be supportable given the mobile environment of the near future. Therefore, in this study, an additional experiment was done to check the sensitivity of the radar sensor. Case studies were performed to confirm the feasibility of the usability of the radar sensor. When the radar was located within 1 m of a subject, there was no significant difference. Furthermore, the second case study showed that the detection of the respiration rate was feasible and relatively accurate when movement did not occur. The proposed method used in this study failed to detect the respiration rate when subjects moved substantially. Therefore, it must be determined how to cancel signal noise due to substantial movements in a future study.

## 5. Conclusions

The most representative method of respiration rate measurement is the contact method using a respiration belt, which is inconvenient to use. In this study, a radar sensor was used to detect the respiration rate in a non-contact manner. Because the radar sensor was sensitive to external noise, the accuracy of the respiration rate detection through peak counting was low even after pre-processing. Therefore, the respiration rate was detected using harmonic quefrency selection in the respiration adaptive domain. This was made possible using the nature of respiration signals. To verify the superiority of the proposed method, the peak counting of the respiration belt and the adaptive harmonic quefrency selection were compared to see whether there was any difference in performance between the respiration belt and the radar sensor. There was none. Furthermore, case studies on changes in the radar position and respiration measurement over extended periods also supported the feasibility of the proposed method.

## Figures and Tables

**Table 1:** Respiration rate result comparison between the respiration belt and radar.

No.	Respiration Rate Comparison			Bland–Altman	
	Belt (Mean ± SD)	Radar (Mean ± SD)	Diff	Mean Difference	95% Limits of Agreement
1	18 ± 0.2	18 ± 0.3	0	−0.1	1.30 (0.5 to −0.8)
2	18 ± 0.3	18 ± 0.3	0	−0.1	1.30 (0.5 to −0.8)
3	19 ± 0.3	19 ± 0.1	0	−0.1	1.30 (0.5 to −0.8)
4	20 ± 0.4	20 ± 0.3	0	0.1	2.36 (1.3 to −1.1)
5	20 ± 0.7	19 ± 0.3	1	−0.6	2.42 (0.6 to −1.8)
6	16 ± 0.2	15 ± 0.1	1	−1.3	2.30 (−0.1 to −2.4)
7	18 ± 0.3	16 ± 0.2	2	−1.4	1.98 (−0.4 to −2.4)
8	20 ± 0.5	18 ± 0.2	2	−1.5	3.14 (0.0 to −3.1)
9	21 ± 0.4	20 ± 0.5	1	−1.4	1.98 (−0.4 to −2.4)
10	19 ± 0.3	18 ± 0.5	1	−1.2	2.86 (0.3 to −2.6)
11	18 ± 0.5	16 ± 0.4	2	−2.1	3.24 (−0.4 to −3.7)
12	20 ± 0.5	20 ± 0.6	0	−0.2	3.26 (1.4 to −1.9)
13	19 ± 1.0	18 ± 1.0	1	−1.1	4.70 (1.3 to −3.4)
14	20 ± 0.6	19 ± 0.4	1	−0.9	3.06 (0.7 to −2.4)
15	20 ± 0.7	22 ± 0.9	2	1.8	2.86 (3.3 to 0.4)
16	20 ± 0.6	20 ± 0.8	0	0.2	3.72 (2.0 to −1.7)

**Table 2:** Respiration rate results between peak counting or FFT and the ground truth.

No.	Respiration Rate Comparison				Bland–Altman			
	Peak Counting		FFT		Peak Counting		FFT	
	Mean ± SD	Diff	Mean ± SD	Diff	M.D.	2SD	M.D.	2SD
1	19 ± 0.4	1	19 ± 0.3	1	0.7	1.94 (1.6 to −0.3)	0.9	1.30 (1.5 to 0.2)
2	19 ± 0.2	1	19 ± 0.3	1	1.1	0.96 (1.5 to 0.6)	0.9	1.30 (1.5 to 0.2)
3	19 ± 0.3	0	20 ± 0.3	1	0.2	1.54 (0.9 to −0.6)	0.5	2.02 (1.5 to −0.5)
4	21 ± 0.5	1	19 ± 1.0	1	1	3.40 (2.7 to −0.7)	−0.9	4.90 (1.5 to −3.4)
5	22 ± 0.4	2	18 ± 2.9	2	2.4	3.66 (4.2 to 0.5)	−1.7	9.58 (3.1 to −6.5)
6	22 ± 0.6	6	17 ± 0.1	1	5.7	2.38 (6.8 to 4.5)	0.3	1.94 (1.3 to −0.6)
7	22 ± 0.7	4	18 ± 0.2	0	4.5	3.94 (6.4 to 2.5)	0.1	2.74 (1.5 to −1.3)
8	19 ± 0.3	1	15 ± 2.6	5	−0.7	2.76 (0.7 to −2.0)	−4.9	11.12 (0.6 to −10.5)
9	21 ± 0.4	0	17 ± 1.4	4	−0.4	1.98 (0.6 to −1.4)	−4.1	5.96 (−1.1 to −7.0)
10	19 ± 0.8	0	17 ± 1.7	2	0.2	3.54 (2.0 to −1.5)	−2.1	7.90 (1.9~−6.0)
11	19 ± 0.8	1	17 ± 0.8	1	0.5	2.02 (1.5 to −0.5)	−0.8	3.98 (1.2 to −2.8)
12	22 ± 0.4	2	21 ± 1.4	1	1.9	2.58 (3.2 to 0.6)	0.8	5.58 (3.6 to −2.0)
13	22 ± 0.3	3	16 ± 2.8	3	3.2	5.58 (6.0 to 0.4)	−3.2	13.18 (3.3 to −9.8)
14	22 ± 0.6	2	16 ± 2.3	4	1.9	2.74 (3.3 to 0.5)	−4.2	8.22 (−0.1 to −8.3)
15	23 ± 0.3	3	18 ± 1.2	2	2.6	3.90 (4.6 to 0.7)	−1.9	6.56 (1.3 to −5.2)
16	22 ± 1.0	2	16 ± 2.5	4	2.5	4.18 (4.6 to 0.4)	−3.6	10.08 (1.4 to −8.7)

**Table 3:** Respiration rate results between two radars and the ground truth.

No.	G.T.	Respiration Rate Comparison				Bland–Altman			
		Radar 1		Radar 2		Radar 1		Radar 2	
	Mean ± SD	Mean ± SD	Diff	Mean ± SD	Diff	M.D.	2SD	M.D.	2SD
1	18 ± 0.2	18 ± 0.3	0	18 ± 0.3	0	0.2	1.54 (0.9 to −0.6)	0.5	2.02 (1.5 to −0.5)
2	18 ± 0.3	18 ± 0.4	0	18 ± 0.3	0	0	2.40 (1.2 to −1.2)	0.1	1.90 (1.1 to −0.8)
3	19 ± 0.2	19 ± 0.2	0	19 ± 0.1	0	0.2	2.08 (1.2 to −0.9)	0.2	2.20 (1.3 to −0.9)
4	20 ± 0.3	19 ± 0.3	1	20 ± 0.4	0	−0.3	2.30 (0.9 to −1.4)	−0.1	1.68 (0.8 to −0.9)
5	18 ± 0.6	18 ± 0.7	0	19 ± 0.3	1	0.3	2.38 (1.5 to −0.8)	0.8	2.20 (1.9 to −0.3)
6	15 ± 0.2	15 ± 0.3	0	15 ± 0.3	0	−0.1	0.96 (0.4 to −0.5)	0.3	1.94 (1.3 to −0.6)
7	18 ± 0.2	18 ± 0.2	0	19 ± 0.3	1	−0.3	1.84 (0.6 to −1.2)	0.3	1.84 (1.2 to −0.6)
8	18 ± 0.2	18 ± 0.1	0	18 ± 0.3	0	−0.5	2.02 (0.5 to −1.5)	−0.1	2.74 (1.3 to −1.5)
9	19 ± 0.3	19 ± 0.4	0	19 ± 0.3	0	0.3	1.84 (1.2 to −0.6)	0.1	1.30 (0.8 to −0.5)
10	18 ± 0.7	18 ± 0.5	0	18 ± 0.6	0	0.1	2.36 (1.3 to −1.1)	0.3	3.38 (2.0 to −1.3)

## References

[1] Al-Khalidi F.Q., Saatchi R., Burke D., Elphick H., Tan S. (2011). Respiration rate monitoring methods: A review. Pediatric Pulmonol..

[2] Miwa H., Sakai K. Development of heart rate and respiration rate measurement system using body-sound. Proceedings of the 2009 9th International Conference on Information Technology and Applications in Biomedicine.

[3] Folke M., Cernerud L., Ekström M., Hök B. (2003). Critical review of non-invasive respiratory monitoring in medical care. Med Biol. Eng. Comput..

[4] Nam Y., Kim Y., Lee J. (2016). Sleep monitoring based on a tri-axial accelerometer and a pressure sensor. Sensors.

[5] Li C., Peng Z., Huang T., Fan T., Wang F., Horng T., Muñoz-Ferreras J., Gómez-García R., Ran L., Lin J. (2017). A review on recent progress of portable short-range noncontact microwave radar systems. IEEE Trans. Microw. Theory Tech..

[6] Ren L., Wang H., Naishadham K., Liu Q., Fathy A.E. Non-invasive detection of cardiac and respiratory rates from stepped frequency continuous wave radar measurements using the state space method. Proceedings of the 2015 IEEE MTT-S International Microwave Symposium.

[7] Zakrzewski M., Raittinen H., Vanhala J. (2011). Comparison of center estimation algorithms for heart and respiration monitoring with microwave Doppler radar. IEEE Sens. J..

[8] Norton M.P., Karczub D.G. (2003). Fundamentals of Noise and Vibration Analysis for Engineers.

[9] Heo H., Sung D., Lee K. Note onset detection based on harmonic cepstrum regularity. Proceedings of the 2013 IEEE International Conference on Multimedia and Expo. (ICME).

[10] Skowronski M.D., Shrivastav R., Hunter E.J. (2015). Cepstral peak sensitivity: A theoretic analysis and comparison of several implementations. J. Voice.

[11] Konstantin-Hansen H., Herlufsen H. (2010). Envelope and cepstrum analyses for machinery fault identification. Sound Vib..

[12] Respiration Recording, Biopac. https://www.biopac.com/knowledge-base/respiration-recording.

[13] Bland J.M., Altman D.G. (1986). Statistical methods for assessing agreement between two methods of clinical measurement. Lancet.

[14] Hanneman S.K. (2008). Design, analysis, and interpretation of method-comparison studies. AACN Adv. Crit. Care.

[15] Kim T.H. (2009). Differential diagnosis and treatment of dyspnea. Korean J. Med..

[16] Sarkar S., Amelung P.J. (2006). Evaluation of the dyspneic patient in the office. Prim. Care Clin. Off. Pract..

[17] Depaso W.J., Winterbauer R.H., Lusk A.A., Dreis D.P., Springmeyer S.C. (1991). Chronic dyspnea unexplained by history, physical examination, chest roentgenogram, and spirometry: Analysis of a seven-year experience. Chest.

[18] Novak P., Novak V. (1993). Time/frequency mapping of the heart rate, blood pressure and respiratory signals. Med. Biol. Eng. Comput..

